# Cortical thinning over two years after first-episode psychosis depends on age of onset

**DOI:** 10.1038/s41537-021-00196-7

**Published:** 2022-03-11

**Authors:** Laura Pina-Camacho, Kenia Martinez, Covadonga M. Diaz-Caneja, Gisela Mezquida, Manuel J. Cuesta, Carmen Moreno, Silvia Amoretti, Ana González-Pinto, Celso Arango, Eduard Vieta, Josefina Castro-Fornieles, Antonio Lobo, David Fraguas, Miguel Bernardo, Joost Janssen, Mara Parellada, PEPs Group, Santiago Madero, Marta Gómez-Ramiro, Elisa Rodriguez-Toscano, Javier Santonja, Iñaki Zorrilla, Itxaso González-Ortega, Nicolás Fayed, Javier Santabárbara, Daniel Berge, Alba Toll, Juan Nacher, Gracián García Martí, Maria Sague-Vilavella, Jose Sanchez-Moreno, Elena de la Serna, Immaculada Baeza, Cristina Saiz-Masvidal, Fernando Contreras, Leticia González-Blanco, Teresa Bobes-Bascarán, Mónica Dompablo, Roberto Rodriguez-Jimenez, Judith Usall, Anna Butjosa, Edith Pomarol-Clotet, Salvador Sarró

**Affiliations:** 1 Department of Child and Adolescent Psychiatry, Institute of Psychiatry and Mental Health, Hospital General Universitario Gregorio Marañón, IiSGM, CIBERSAM, School of Medicine, Universidad Complutense, Madrid, Spain; 2 Department of Child and Adolescent Psychiatry, Institute of Psychiatry, Psychology and Neuroscience, King’s College London, London, UK; 3 Barcelona Clinic Schizophrenia Unit, Hospital Clinic of Barcelona, Neuroscience Institute; Department of Medicine, Institut de Neurociències, Universitat de Barcelona; CIBERSAM; August Pi I Sunyer Biomedical Research Institute (IDIBAPS), Barcelona, Spain; 4 Psychiatry Department, Complejo Hospitalario de Navarra, Pamplona, Spain Navarra Institute for Health Research (IdiSNA), Pamplona, Spain; 5 Department of Psychiatry, School of Medicine, University Hospital of Alava-Santiago, CIBERSAM, University of the Basque Country, Vitoria-Gasteiz, Spain; 6 Barcelona Bipolar Disorders Program, Hospital Clinic, University of Barcelona, IDIBAPS, CIBERSAM, Barcelona, Spain; 7 Department of Child and Adolescent Psychiatry and Psychology, Institut Clinic de Neurociències, 2017SGR881, Hospital Clínic Universitari, CIBERSAM, Barcelona, Spain; 8 Department of Medicine and Psychiatry, Universidad de Zaragoza, Instituto de Investigación Sanitaria Aragón (IIS Aragón), CIBERSAM, Zaragoza, Spain; 9 Institute of Psychiatry and Mental Health, Hospital Clínico San Carlos, IdISSC, CIBERSAM, School of medicine, Universidad Complutense, Madrid, Spain; 10 Barcelona Clinic Schizophrenia Unit, Hospital Clinic of Barcelona, Neuroscience Institute, CIBERSAM, Barcelona, Spain; 11 Department of Child and Adolescent Psychiatry, Institute of Psychatry and Mental Health, Hospital General Universitario Gregorio Marañon, CIBERSAM, IiSGM,School of Medicine, Universidad Complutense, Madrid, Spain; 12 Department of Psychiatry, University Hospital of Alava-Santiago, CIBERSAM, School of Medicine, University of the Basque Country, Vitoria-Gasteiz, Spain; 13 Unidad de Neurorradiología, Hospital Quironsalud, Instituto de Investigación Sanitaria IIS) Aragón, Zaragoza, Spain; 14 Department of Preventive Medicine and Public Health, Universidad de Zaragoza, Instituto de Investigación Sanitaria IIS) Aragón, CIBERSAM, Zaragoza, Spain; 15 Hospital del Mar Medical Research Institute, CIBERSAM, Autonomous University of Barcelona, Barcelona, Spain; 16 Neurobiology Unit, Program in Neurosciences and Interdisciplinary Research Structure for Biotechnology and Biomedicine (BIOTECMED), Universitat de València, Burjassot, CIBERSAM: Research Institute of Clinic University Hospital of Valencia (INCLIVA), Valencia, Spain; 17 Radiology Department, Quirónsalud Hospital, CIBERSAM, Valencia, Spain; 18 Department of Child and Adolescent Psychiatry and Psychology, Neuroscience Institute, Hospital Clínic de Barcelona,Department of Medicine, University of Barcelona, 2017SGR881, CIBERSAM, Barcelona, Spain; 19 Bellvitge Biomedical Research Institute IDIBELL; Department of Psychiatry- Bellvitge University Hospital, Hospitalet de Llobregat- Barcelona, Spain; University of Barcelona, Department of Clinical Sciences- School of Medicine, Barcelona, Spain; 20 Servicio de Salud del Principado de Asturias -SESPA-, Universidad de Oviedo, Instituto de Investigación Sanitaria del Principado de Asturias –ISPA, CIBERSAM, Oviedo, Spain; 21 Instituto de Investigación Sanitaria Hospital 12 de Octubre IMAS12), CIBERSAM, CogPsy Group, Universidad Complutense, Madrid, Spain; 22 Parc Sanitari Sant Joan de Déu, Research and Development Unit, Sant Boi de Llobregat, Barcelona, Spain; 23 FIDMAG Germanes Hospitalàries Research Foundation, CIBERSAM, Barcelona, School of Medicine, Universitat Internacional de Catalunya, Barcelona, Spain

**Keywords:** Psychosis, Biomarkers, Neuroscience

## Abstract

First-episode psychosis (FEP) patients show structural brain abnormalities at the first episode. Whether the cortical changes that follow a FEP are progressive and whether age at onset modulates these changes remains unclear. This is a multicenter MRI study in a deeply phenotyped sample of 74 FEP patients with a wide age range at onset (15–35 years) and 64 neurotypical healthy controls (HC). All participants underwent two MRI scans with a 2-year follow-up interval. We computed the longitudinal percentage of change (PC) for cortical thickness (CT), surface area (CSA) and volume (CV) for frontal, temporal, parietal and occipital lobes. We used general linear models to assess group differences in PC as a function of age at FEP. We conducted post-hoc analyses for metrics where PC differed as a function of age at onset. We found a significant age-by-diagnosis interaction effect for PC of temporal lobe CT (*d* = 0.54; *p* = 002). In a post-hoc-analysis, adolescent-onset (≤19 y) FEP showed more severe longitudinal cortical thinning in the temporal lobe than adolescent HC. We did not find this difference in adult-onset FEP compared to adult HC. Our study suggests that, in individuals with psychosis, CT changes that follow the FEP are dependent on the age at first episode, with those with an earlier onset showing more pronounced cortical thinning in the temporal lobe.

## Introduction

Studies assessing brain cortical changes in the first phases of psychotic disorders are important for understanding the neurobiological underpinnings of the illness. Converging evidence supports that structural brain abnormalities present at the first episode of psychosis (FEP) are the consequence of years-long deviant developmental processes that begin long before the first manifestations of psychosis emerge^1,2^. Some of these abnormalities seem to progress during the initial years and persist well into the more chronic phases of psychosis^3,4^. However, whether the changes are progressive or not remains unclear, and results from longitudinal magnetic resonance imaging studies are mixed^5^. Furthermore, age at onset has been associated with the gray matter^6,7^ and white matter abnormalities^8^ that FEP patients display at the first episode. Using a cross-sectional design, we (the PEPs group) have previously reported that age at FEP onset modulates the diagnostic-related cortical abnormalities that patients aged 12–35 years displayed at the first episode^6^. In that study we found that patients with adolescent onset of schizophrenia spectrum disorders (but not those with an adult onset) showed cortical volume and thickness deficits principally in frontal and temporal regions, which are regions reportedly undergoing active maturation at that developmental stage. Yet, it is still not clear whether age at onset modulates the presence and severity of longitudinal cortical changes that occur after first-episode onset. To our knowledge, no study has yet included a combined sample of adolescents and adults to specifically assess the association between age at FEP onset and the cortical changes that follow a FEP.

In the present study, we aimed to determine whether the cortical thickness, volume and surface area abnormalities present at the FEP evolve over the first two years of the illness, and, if so, whether age at FEP onset modulates the observed change trajectories. Based on our previous study^6^, we hypothesized that, relative to a sample of age-matched neurotypical healthy controls (HC), FEP individuals would show progressive cortical thinning and volume loss of frontal and temporal regions, with adolescent-onset cases (relative to adult-onset cases) showing more pronounced changes. As a secondary objective, we aimed to describe the clinical characteristics of a biologically-driven FEP cluster that shows the most pronounced age-related longitudinal cortical changes.

## Results

### PEPs-Img-long sample description

Table 1 shows descriptives for FEP and HC clinical and demographic variables. No differences were found between groups in any of these variables (all *p* > 0.05), except for a lower premorbid IQ in the FEP group (*d* = 1.003; *p* < 0.001).

**Table 1:** Clinical and demographic descriptives for FEP and HC groups.

	FEP (*N* = 74)	HC (*N* = 64)	Cohen’s *d*; *p* value
Site, No (%)^1^			
Barcelona	40 (54.1)	35 (54.7)	*d* = 0.013; *p* = 0.941
Madrid	11 (14.9)	12 (18.8)	*d* = 0.104; *p* = 0.541
Zaragoza	15 (20.3)	11 (17.2)	*d* = 0.079; *p* = 0.644
Vitoria	8 (10.8)	6 (9.4)	*d* = 0.048; *p* = 0.781
Age at T1 scan. Years, mean (SD) [range]	23.2 (6.00) [15–35]	23.7 (5.92) [15–35]	*d* = 0.096; *p* = 0.575
Sex. Male, No. (%)	50 (67.6)	43 (67.2)	*d* = 0.008; *p* = 0.962
Illness duration. Days, median, IQR = 3	117 [76–231]	--	--
Time between T1 and T2 scans. Months, mean (SD) [range]	24.5 (2.5) [16–31]	23.9 (2.8) [20–32]	*d* = 0.229; *p* = 0.182
Ethnicity, No. (%).			
Caucasian	68 (91.9)	58 (90.6)	*d* = 0.048; *p* = 0.792
Hispanic	3 (4.1)	3 (4.7)	
Other	3 (4.1)	3 (4.7)	
Handedness, No. (%)			
Right-handed	59 (79.7)	53 (82.8)	*d* = 0.079; *p* = 0.644
Left-handed	8 (10.8)	7 (10.9)	*d* = 0.005; *p* = 0.981
Mixed	4 (5.4)	2 (3.1)	*d* = 0.112; *p* = 0.686
Parental socioeconomic status^2^ No. (%)			
High – intermediate (vs. low)	53 (71.6)	54 (84.4)	*d* = 0.308; *p* = 0.073
Premorbid IQ.^3^ Score, mean (SD) [range]	93.8 (16.9) [60–140]	108.3 (11.9) [85–145]	*d* = 1.003; *p* < 0.001
Diagnostic subgroup^4^, No. (%)		--	--
SSD	38 (51.4)		
AFP	17 (23.0)		
Other psychoses	19 (25.7)		
Illness severity (CGI-S) at T1, mean (SD)	4.40 (1.3)	--	--
CGAS/GAF score at T1, mean (SD)	53.5 (23.1)	93.3 (5.7)	*d* = 2.284; *p* < 0.001
Cumulative antipsychotic dose at T2^5^, mg, median [range]	301.042 [0–1.088.332]	--	--

In all cells, % refers to percentages (within columns) of participants for whom information was available. For qualitative variables, Chi-square (χ2) or Fisher tests were used. For quantitative variables, *t* tests or Mann–Whitney *U* tests were used.

*CGAS* Children’s Global Assessment Scale, *CGI-S* Clinical Global impression-Severity Scale, *FEP* first episode of psychosis, *GAF* Global Assessment of Functioning Scale, *HC* healthy control, *IQ* intelligence quotient, *T1* baseline scan visit, T2 follow-up scan visit.

^1^Note: two sites of the PEPs-Imaging cross-sectional study did not participate in the longitudinal branch of the study.

^2^Socioeconomic status (SES) defined with the Hollingshead-Redlich scale. Individuals were categorized into a low (SES 4–5) vs. intermediate-high (SES 1–3) parental SES subgroup.

^3^Estimated with the vocabulary subtest of the WISC-IV or WAIS-III for subjects under and over 16 years of age.

^4^SSD: 24-month follow-up diagnosis of schizophrenia spectrum disorder (schizophrenia, schizophreniform, or schizoaffective disorder); AFP: 24-month follow-up diagnosis of affective psychosis (type I bipolar disorder or major depressive disorder with psychotic symptoms); Other psychoses: 24-month follow-up diagnosis of brief reactive psychosis, delusional disorder, substance-induced psychotic disorder, and psychotic disorder not otherwise specified.

^5^In chlorpromazine equivalents: 100 mg chlorpromazine = about 1.5 mg risperidone/5 mg olanzapine/150 mg quetiapine.

Supplementary Table 1 shows descriptives for FEP and HC morphometric variables (i.e. lobar CT/CSA/CV at T1, at T2 and PC of CT, CSA and CV). There was no effect of diagnosis (FEP vs. HC) nor of age at baseline scan in any of these metrics, all corrected *p* values (36 tests) > 0.001.

### Main analysis <whole sample>. The effect of age-by-diagnosis on CT/CSA/CV longitudinal changes at lobar level

**Fig. 1:**
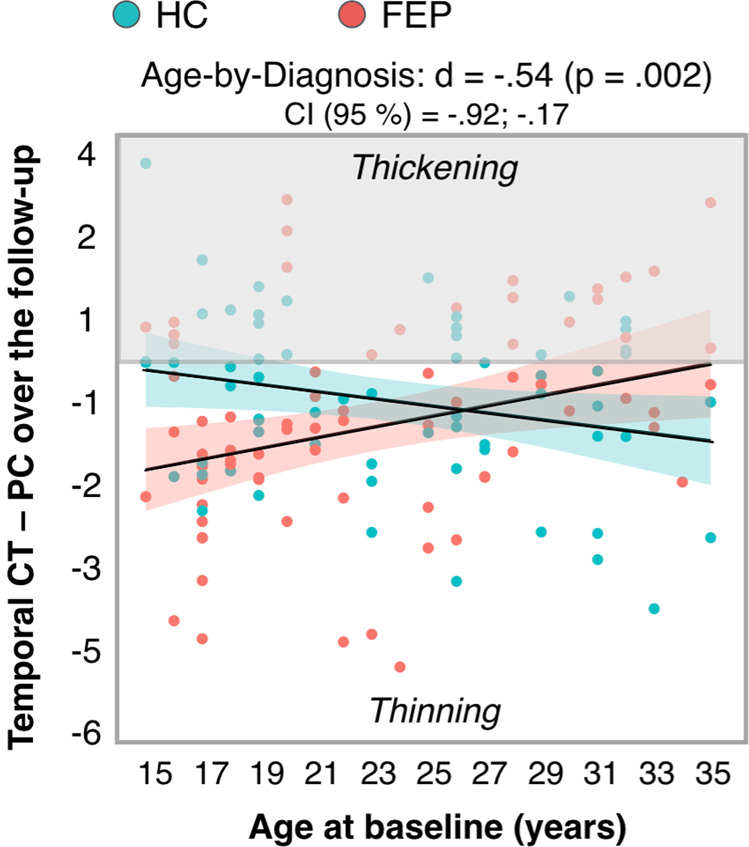
Age-by-diagnosis interaction effect on PC of temporal lobe CT. For FEP and HC, PC values < 0 indicate cortical thinning (CT decrease) over follow-up.; PC values > 0 indicate thickening (CT increase) over follow-up. Abbreviations: CT cortical thickness, FEP first episode psychosis, HC healthy controls, PC percentage of change.

At a lobar level, we only found a significant age-by-diagnosis effect on the PC of temporal lobe CT (*d* = −0.54, corrected *p* value (12 tests) = 0.002) (Fig. 1), with younger (but not older) FEP patients showing significant cortical thinning, relative to HC.

**Fig. 2:**
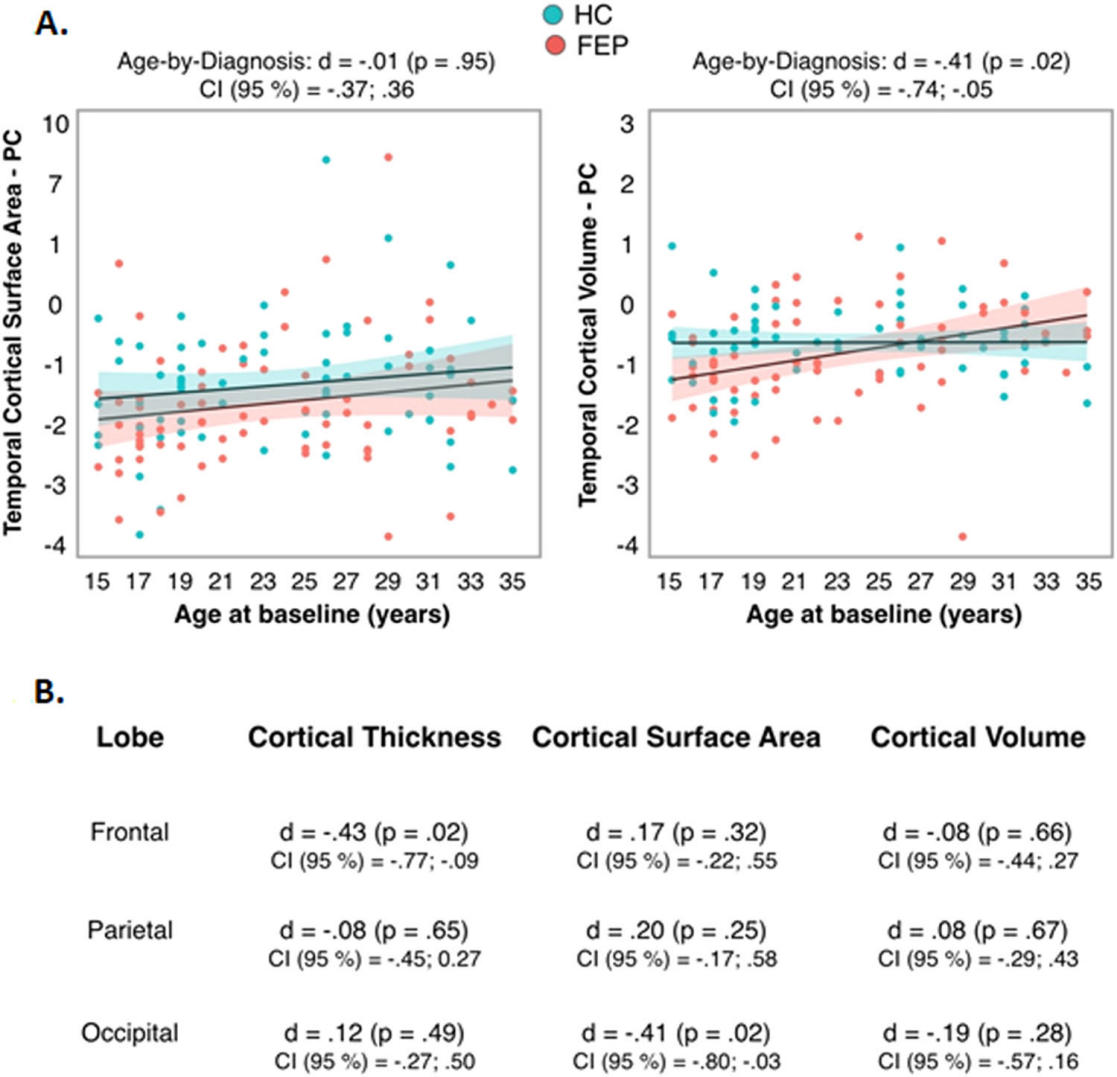
Age-by-diagnosis interaction effect on PC of temporal lobe CSA/CV and on PC of frontal, parietal and occipital CT/CSA/CV. **A** Age-by-diagnosis interaction effect on PC of temporal lobe CSA (left side) and CV (right side). For FEP and HC, PC values < 0 indicate decrease (in surface area or volume) over follow-up.; PC values > 0 indicate increase (in surface area or volume) over follow-up. **B** Cohen’s d, *p* values and confidence intervals (CI, 95%) for the age-by-diagnosis interaction effect on PC of frontal, parietal and occipital CT/CSA/CV. Abbreviations: CSA cortical surface area, CV cortical volume, FEP first episode psychosis, HC healthy controls, PC percentage of change.

Conversely, no significant age-by-diagnosis effects were found for PC of CSA/CV in the temporal lobe (Fig. 2a), nor for PC of frontal, parietal or occipital CT/CSA/CV (Fig. 2b), all corrected *p* values (12 tests) > 0.004.

### Secondary analysis 1 <whole sample>. The effect of age-by-diagnosis on CT/CSA/CV longitudinal changes in temporal lobe regions

At a regional level, the largest age-by-diagnosis effects were for PC of middle (*d* = −0.43, *p* = 0.01) and inferior (*d* = −0.48, *p* = 0.007) temporal gyri CT.

### Secondary analysis 2 <whole sample>. The effect of diagnosis on temporal lobe CT at T1 and T2: separately for the adolescent FEP-HC, and adult FEP-HC samples

As described in “Methods”, we used a cutoff of 19 years to define a “≤19 year” (adolescent) FEP-HC group (*n* = 26 FEP, 24 HC) and a “>19 year” (adult) FEP-HC group (*n* = 48 FEP, 40 HC). Demographic and clinical characteristics of these groups are presented in Supplementary Table 2. One of the sites had more FEP patients in the adolescent group, while another site had only adult patients. Within the adolescent FEP group, mean IQ was 10 points lower, there were more ´other psychosis´ diagnoses, the severity of the illness was greater, and the global functioning lower.

**Fig. 3:**
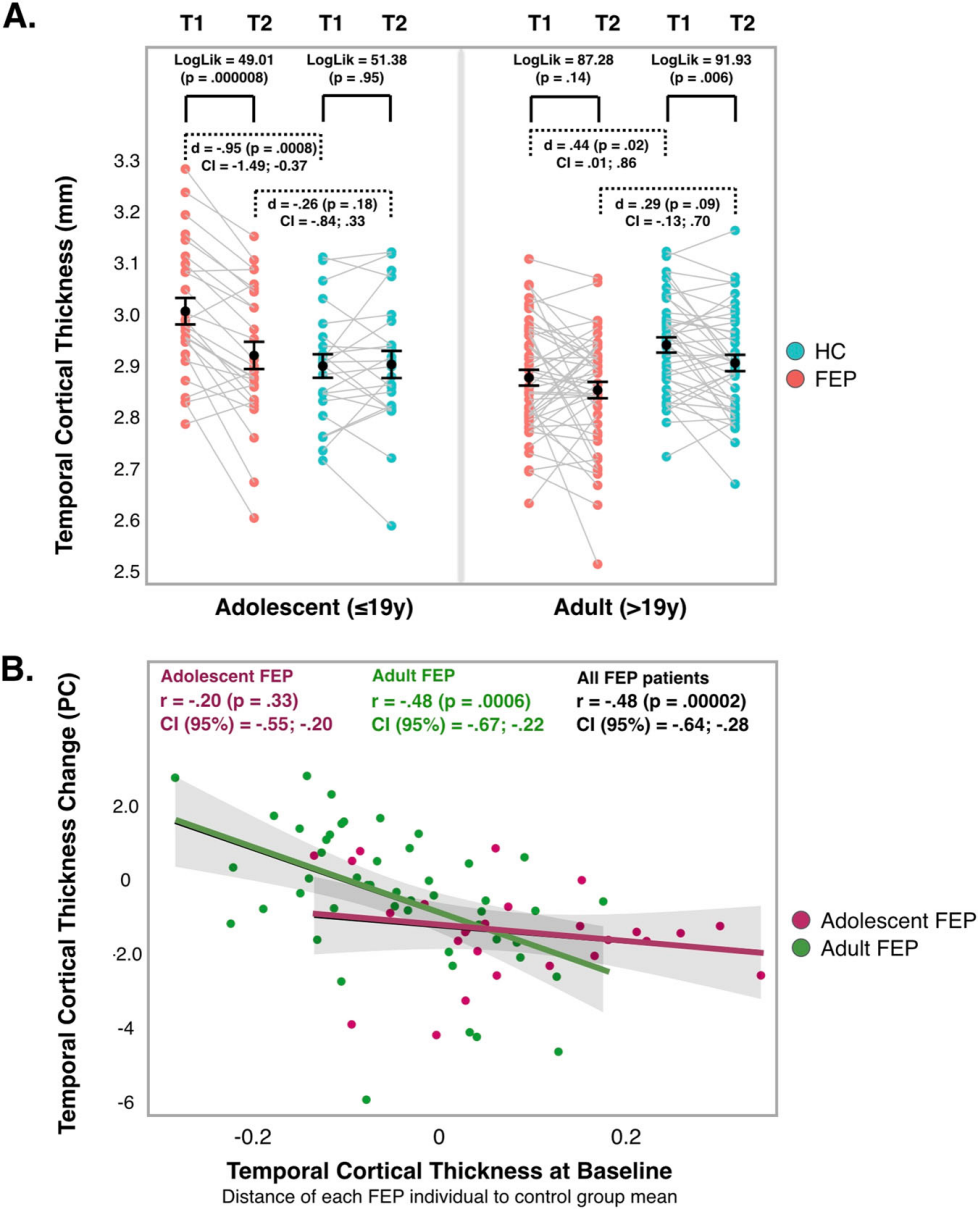
Temporal lobe CT for adolescent and adult FEP and HC. **A** Temporal lobe CT for adolescent and adult FEP and HC at T1 and T2. Dashed brackets: GLMs (FEP vs. HC comparisons, at T1 and T2). Solid brackets: Repeated measures mixed models (intragroup T2–T1 change of temporal lobe CT). **B** Association between temporal lobe CT at T1 and PC of temporal lobe CT, for adolescent FEP, adult FEP, and whole FEP samples. Pearson’s correlations, *p* values and confidence intervals (95%) shown. “*x*” axis: CT values > 0 indicate thicker cortex in FEP relative to HC, CT values < 0 indicate thinner cortex. “*y* axis”: PC values < 0 indicate cortical thinning; PC values > 0 indicate cortical thickening over follow-up. T1: baseline scan; T2: follow-up scan. CT cortical thickness, FEP first-episode psychosis, HC healthy controls, PC percentage of change.

Figure 3a shows the dispersion of temporal lobe CT at T1 and T2 separately for the adolescent FEP-HC, and adult FEP-HC samples. Regarding FEP vs. HC differences, both the adolescent and the adult FEP group showed higher mean CT at T1 [*d* = −0.95 (*p* = 0.0008) for adolescents; *d* = 0.44 (*p* = 0.02) for adults, respectively], a difference that was no longer present at T2 (Fig. 3a), adolescent and adult section, red dots vs. blue dots. In the linear mixed models, we found a significant decrease (T2-T1) in temporal lobe CT in the adolescent FEP (LogLik = 49.01, *p* = 0.000008) (Fig. 3a, adolescent section, red dots T1 vs. red dots T2) and the adult HC groups (LogLik = 91.93, *p* = 0.006) (Fig. 3a, adult section, blue dots T1 vs. blue dots T2).

### Secondary analysis 3 <FEP sample>. Exploring the FEP group (adolescent or adult) whose temporal lobe CT measurements changed most over the follow-up period

As shown in Fig. 3b, among adolescent FEP patients (purple dots), those showing the largest “temporal lobe CT at T1 patient-control distance” (*x* axis > 0) were those showing a trajectory of greater temporal lobe cortical thinning (PC decrease) over the follow-up period (*y* axis < 0).

### Secondary analysis 4 <FEP sample>. Clustering of FEP patients based on their temporal lobar and regional CT

The optimal number of clusters found in the morphometric cluster analysis of FEP patients was two: cluster 1, *n* = 43, and cluster 2, *n* = 31. Supplementary Table 3 shows descriptives and differences between cluster 1 and 2 in temporal lobar and regional CT at T1 and PC. Relative to cluster 1 patients, cluster 2 patients had thicker cortex in the temporal lobe, and in the middle, inferior, fusiforrm and parahippocampal gyri at T1 (all *p* < 0.05), alongside greater cortical thinning of all the studied temporal regions over the follow-up period (except for the transverse temporal), all *p* < 0.001 (see Supplementary Table 3). Repeated measures linear mixed models also revealed that cluster 2 patients showed a significant longitudinal (T2–T1) decrease in the CT of all studied temporal regions, all *p* < 0.05, which was not found in cluster 1 patients (Supplementary Table 4).

FEP patients belonging to cluster 2 were younger (*d* = 0.591, *p* = 0.014) and received lower dose of antipsychotics over the follow-up period (*d* = 0.577, *p* = 0.016). Cluster 2 also had a higher proportion of adolescent-onset patients (45.2% vs. 27.9%), although this difference was not significant (*p* = 0.125). We found no other differences between cluster 1 and 2 patients in their demographic or clinical baseline or follow-up characteristics (Supplementary Table 5).

**Fig. 4:**
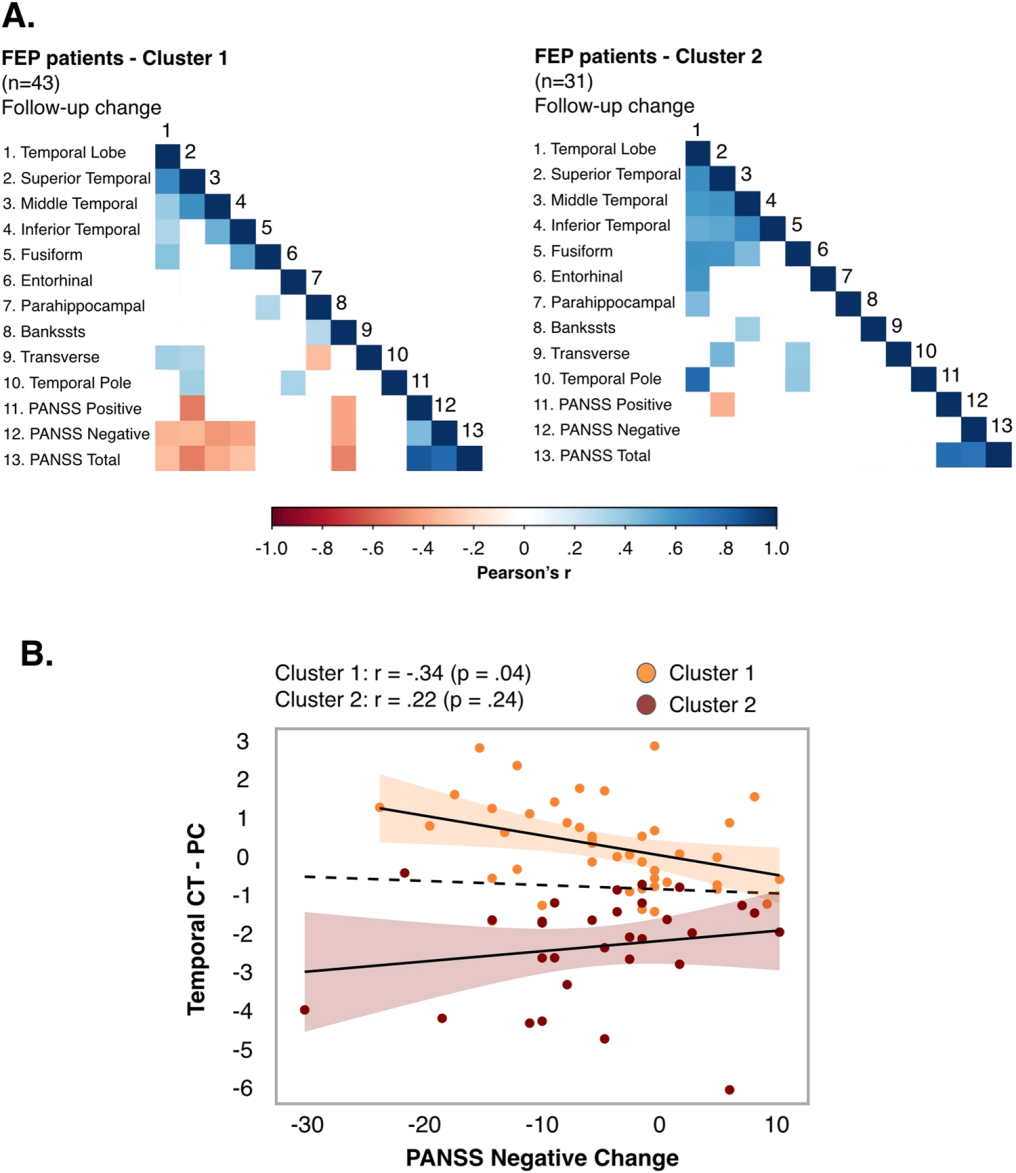
Association between PC of temporal lobe CT and symptom change scores in FEP clusters 1 and 2. **A** Association between PC of temporal lobe CT (at lobar and regional level) and PANSS positive, negative, and total change scores, in FEP clusters 1 and 2. In the correlation matrix, only significant relationships (*p* < 0.05) are color-coded according to the color bar on the bottom. **B** Association between PC of temporal lobe CT and PANSS negative change score, in FEP clusters 1 and 2. Pearson’s correlations, *p* values and confidence intervals (95%) shown for FEP clusters 1 and 2. “*x* axis”: PANSS change scores < 0 indicate symptom improvement, PANSS change scores > 0 indicate symptom worsening over follow-up. “*y* axis”: PC values < 0 indicate cortical thinning; PC values > 0 indicate cortical thickening over follow-up. Abbreviations: CT cortical thickness, FEP first-episode psychosis, PANSS positive and negative syndrome scale, PC percentage of change.

Figure 4a shows, separately for cluster 1 and cluster 2 patient groups, the association between temporal lobar and regional PC of CT and patients’ PANSS positive, negative and total two-year change scores. Figure 4b (for the temporal lobe) and Supplementary Fig. 1 (for four temporal regions) show, in particular, the association between PC of CT in those regions and patient’s PANSS negative two-year change score. For cluster 1 patients, the smaller the PC of CT in superior, middle and inferior temporal gyri, the smaller the improvement in PANSS negative score over time.

Supplementary Fig. 2 shows the association between temporal lobar and regional PC of CT and other clinical variables of interest. For cluster 1 patients, CGAS/GAF scores at follow-up were positively correlated with PC of temporal lobe CT (*p* < 0.05), meaning that the smaller the CT change over the follow-up period, the worse the individual’s functioning at follow-up. Note that cluster 1 patients as a whole did not show significant longitudinal change (T2–T1) in temporal lobar and regional CT over the follow-up period (see previous section).

## Discussion

In this study we assessed the association of age at first episode psychosis and the changes in volume, surface area and thickness in the two years that follow a FEP in a sample of patients whose psychotic onset ranges from adolescence through adulthood (15–35 years). We found that age at first episode was associated with the dynamics of the observed longitudinal changes, so that only patients with an earlier age at first episode (around 15–19 years) showed longitudinal cortical thinning in the temporal lobe relative to an age-matched sample of healthy controls. Our post-hoc analyses using an age cut-off of 19 years also revealed that longitudinal changes in temporal cortical thickness were only evident for adolescent but not for adult patients. We also found that temporal cortical thinning in the adolescent-onset group was more pronounced (a) in medial and inferior temporal gyri, and (b) in those patients already showing reduced thickness at baseline relative to controls (possibly implying progression of baseline deficits in this FEP subgroup).

Our study presents an advance in that it shows divergent longitudinal brain abnormalities in adolescent- and adult-onset FEP patients relative to healthy controls. Previous longitudinal MRI studies were using age at FEP as a categorical discrimination factor for comparing adolescent^9^ or adult^10^ patients and healthy controls, or were simply relying on narrow age-range samples that do not encompass important stages of brain development. We do not know of a study that had included a combined sample of adolescents and adults (wide age range: 15–35 years) to specifically assess the effect of age of onset on the longitudinal brain changes that follow the first-episode psychosis. Our study demonstrates that in order to further the understanding of the physiopathology of psychotic disorders, a sample comprising the various stages of brain maturation at the first stages of the illness is preferred.

The results from this study also warrant caution when interpreting case-control comparison studies that do not account for age-dependent differential effects on longitudinal trajectories of brain changes, especially when patients with psychotic symptom onset below and over 19 years are included in the same study. In fact, in our study, no differences were found between FEP and HC in any of the lobar measurements at T1 or T2, nor in their PC values when comparing the whole 15–35 year sample.

In keeping with our previous cross-sectional study^6^, our results should be interpreted in the context of what is known about the heterochrony of typical brain maturity, i.e., different structures mature at different ages and rates and differ between individuals^11–13^. If onset of psychosis coincides with active cortical neurodevelopmental changes in a brain structure, then that particular brain structure will be more affected by disease progression after the FEP^6^. In this vein, frontal and occipital poles are likely to be the ones that are most actively maturing during the early adolescent period (13–16 years) and psychosis onset at this stage is associated with greater case-control differences in cortical measures in these lobes^13^. The temporal cortex structures mature later - during late adolescence^13^, and onset of psychosis during this period has been shown to principally affect temporal regions^6,9,14^ as was the case in our study. Along these lines, in our sample we observed the most “extreme” case-control difference in temporal thickness PC at an age cut-off of 19 years. We also found that among adolescent-onset patients, those showing the largest temporal thickness deficits at baseline (relative to their control counterparts) were also those showing a trajectory of greater temporal thinning over the follow-up period. Finally, we found in the cluster analysis that those patients showing greater temporal thinning over the follow-up (cluster 2 patients) were younger than those showing less pronounced or no significant changes (cluster 1 patients).

Another factor to consider is that normal processes of brain maturation leading to temporal thickness decrease over time could be exaggerated in our sample of young patients with psychosis, thus leading to “greater than expected” decreases in cortical measurements, in the absence of neurodegeneration. That said, in our sample, younger patients had greater illness severity and lower IQ scores and received lower doses of antipsychotics over the follow-up period, thus allowing for the possibility that these variables, linked to age at onset, modulated or influenced the reported changes. However, neither IQ nor cumulative dose of antipsychotics showed a significant effect on the longitudinal change of the studied ROIs when included in the statistical models.

Our case-control analysis approach remains focused on group averages (e.g., the “average psychosis patient”) and treats individual differences principally as noise, disregarding larger heterogeneity of structural brain metrics in patients when compared to healthy controls^15^. Objectively defining biological subtypes within the FEP construct based on neuroanatomical data is important for further progress on this topic^16^. In the absence of such definitions, we used a multivariate clustering approach based on a set of “developmentally relevant” structural brain measures (those longitudinal measurements where we found a significant age-by-diagnosis interaction) to identify subgroups of FEP individuals with common biological indicators, i.e., biologically-driven subgroups, rather than phenomenologically-based groups of patients^17^. We found two biologically-driven clusters. Cluster 2 patients had, relative to cluster 1 patients, a thicker temporal lobe at baseline and greater temporal cortical thinning over the follow-up period. Patients in cluster 2 were younger and had a higher (although not statistically significant) proportion of adolescent-onset patients. For cluster 1 patients, CGAS/GAF scores at follow-up were positively correlated with the longitudinal change of temporal thickness, such that the smaller the thickness change over the follow-up period, the worse the individual’s functioning at follow-up. Biologically-based clustering may be of use to constrain the intrinsic biological and clinical heterogeneity of the psychosis construct^17^. Our study adds to previous studies applying a similar approach; that is, using a developmental and longitudinal outlook to select biological variables relevant for clustering. Alternative approaches such as neurodevelopmental normative modeling are also emerging as promising tools in the study of psychotic illness^18^. In this regard, there are collaborative efforts in progress aiming to map normative development across the life span. The logical next step will be to systematically map the heterogeneity of mental disorders across biological readouts^19^.

Results derived from this study should be interpreted in the context of several limitations. First, in this study we used “age at baseline scan” as a proxy for “age at psychosis onset”. Neither age at onset of positive symptoms nor age at scan gives the most accurate estimate of actual psychosis onset. Onset of positive symptoms may not reflect the actual psychotic onset itself and cognitive/negative symptoms are hard to assess accurately using retrospective assessments. Age at baseline scan may, to some extent, reflect the effect of both emergence and progression of psychosis over the initial months of the psychotic break^20^. In this study, we chose age at scan since it is a more objective and reliable measure and relies on the actual neurodevelopmental point in time when patients and controls are imaged. Furthermore, this is a relatively recent-onset sample (median time from positive symptom onset to baseline scan = 117 days) and we did not find any significant association between time from symptom onset to baseline morphometric measurements^6^. Second, the definition of PC implies an assumption that the rate of change over the follow-up period is constant. Third, despite the use of site as a covariate, the use of a data harmonization technique, and conducting a reproducibility study beforehand, it is difficult to argue that we have fully controlled for the effect of site, so we should treat the results with caution. Four, less than half of the PEPs-Img cross sectional sample was included in the PEPs-Img longitudinal study and had complete data, since two of the six sites participating in the PEPs-Img cross-sectional study did not participate in the PEPs-Img longitudinal branch. Previous studies have also found that patients with an early onset of psychosis are difficult to engage and retain in clinical research^21^. Finally, analyses at lobar level were Bonferroni corrected but we did not apply any formal correction for any of the post-hoc analyses at regional or cluster level, as these analyses were all exploratory.

The main strengths of this study include the inclusion of a unique deeply phenotyped sample of FEP patients with a large age span (being the first study to assess a combined sample of adolescents and adults aged 15–35 years), the inclusion of very recent-onset cases, the careful patient-control matching strategy and the handling of potential confounders.

Our study suggests that, in psychosis patients, longitudinal brain changes that follow the first episode are influenced by the individual’s age at first episode, particularly at those ages when the brain is still undergoing major developmental changes (i.e., adolescence). Indeed, our study revealed that during the first two years that follow a FEP, adolescent patients show greater cortical thinning of the temporal lobe than adult patients, relative to their age-matched healthy control counterparts. This highlights the need for longitudinal studies including participants with psychosis across their life spans to further assess abnormal brain developmental trajectories from the very early stages of the disorder, and to advance understanding of the neurobiological underpinnings of these pathological changes.

## Methods

### Study design

The sample was drawn from the PEPs-imaging study, a two-year, multicenter, naturalistic, prospective study, performed in the context of the Spanish CIBERSAM network^22,23^ in which 196 FEP patients and 157 HC were recruited from 2009 to 2011 and scanned at first episode on six scanner platforms^6^. Patients fulfilling the following inclusion criteria were recruited from outpatient and inpatient units: age 7–35 years at the time of study entry, psychotic disorder (according to DSM-IV criteria) of <12 months’ duration, able to read and speak Spanish fluently, and written informed consent. Exclusion criteria included intelligence developmental delay (mental retardation according to DSM-IV criteria), history of head trauma with loss of consciousness, and systemic disease with mental health impact. We recruited HC with negative first-degree family history of psychotic disorder or major depressive disorder from the same geographic areas.

Experienced psychiatrists and psychologists trained in the assessment tools gathered demographic diagnostic, clinical, and functional data at baseline and two-year follow-up timepoints from the team reviewed medical records and conducted interviews with participants and/or parents/legal guardians where appropriate. A complete description of the study design and assessment procedures is available elsewhere^6,24^. The authors assert that all procedures contributing to this work comply with the ethical standards of the relevant national and institutional committees on human experimentation and with the Helsinki Declaration of 1975, as revised in 2008. All procedures involving human subjects/patients were approved by the institutional review boards (IRBs) of all the participant sites (Hospital Clinic, Hospital Bellvitge, Hospital Benito-Menni, Hospital San Joan de Deu, in Barcelona; Hospital Gregorio Marañon in Madrid, Hospital Santiago Apostol in Vitoria, and Instituto Aragones (HCU/HUMS) in Zaragoza). We obtained written informed consent from adult participants, parent and legal guardians, and written assent from minor participants.

### Definition of age at FEP onset

In this study “age at FEP onset” was made comparable to “age at baseline scan” and patients were scanned around the time of positive psychotic symptom onset. Age at scan is an objective and reliable measure, as this is a relatively recent-onset sample (median time from positive symptom onset to baseline scan = 117 days, IQR = 3 [76–231] days^6^).

### Image acquisition and analysis

FEP patients and HC underwent two T1-weighted MRI scans with a 2-year follow-up interval (median interval = 24, IQR = 3 [25–22] months). Supplementary Table 6 shows acquisition parameters by site. We visually inspected the scans for abnormalities and none were found. Akin to the ENIGMA protocol (http://enigma.usc.edu/protocols/imaging-protocols) we excluded subjects with outlier measurements (i.e., *z* scores 3 SD above or below the mean) on 1 or more of the normalized cortical volume labels generated by FreeSurfer by default (66 cortical regions)^25^. This led to the exclusion of 2 FEP patients. To further assess whether subtle variability in image quality was associated with diagnosis we compared the Euler number, a measure of image quality generated by FreeSurfer^26^ between the diagnostic groups (FEP and HC). No significant difference in Euler numbers was found (mean FEP = −85.5 (57.8) vs. HC = −85.5 (54.6), left hemisphere; *p* = 0.599; mean FEP = −81.4 (60.1) vs. HC = −86.1 (64.4), right hemisphere; *p* = 0.659).

We calculated total brain volume (TBV) as the sum of total gray matter and white matter volumes. We used the FreeSurfer analysis suite (v5.3, available at http://surfer.nmr.mgh.harvard.edu/) with default settings to generate, for each time point (baseline-T1 and follow-up-T2), cortical thickness (CT), surface area (CSA) and volume (CV) measurements^27^ for each region of interest (ROI)^25^, including: (i) lobar ROIs (frontal, parietal, temporal, and occipital lobe), and (ii) regional ROIs within each lobe (Supplementary Table 7). All measures were summed or averaged over both hemispheres to provide one measure per ROI. Qualified experts supervised the image processing. We harmonized all data using a technique adopted from genomics: the ComBat algorithm scripted for R following the author’s guidelines. ComBat is an effective harmonization technique that removes unwanted variation associated with site and is adapted for modeling and removing site effects in multi-site imaging studies^28,29^.

After obtaining CT/CSA/CV for each ROI at T1 and T2, we computed the longitudinal percentage of change (hereinafter referred to as PC) as the slope of each particular metric relative to (i.e., adjusted for) its metric at baseline, as follows:

1 $${{{\mathrm{PC}}}} = ((({{{\mathrm{T}}}}2 - {{{\mathrm{T}}}}1))/{{{\mathrm{T}}}}1)/({{{\mathrm{time}}}}\;{{{\mathrm{between}}}}\;{{{\mathrm{scans}}}}({{{\mathrm{months}}}})) \times 100$$

T1 = baseline metric, T2 = follow-up metric

PC represents degree of change over the follow-up period, assuming that it is constant, and provides a percentage value estimate that can be compared across the various metric measurements.

### Participants

The PEPs-imaging-longitudinal study included participants from four sites who completed the baseline and the follow-up scan assessment (76 FEP patients and 67 HC, referred as “completer” group). There was a higher proportion of low socioeconomic status (SES) subjects both in the “completer” FEP and HC groups (all *p* < 0.05). We found no other differences in demographic/clinical variables between “completer” and “non-completer” FEP and HC samples (Supplementary Table 8a), nor in their main global measurements at baseline (Supplementary Table 8b).

Before any analysis, and as mentioned, we excluded *n* = 2 scans from FEP patients with outlier measurements on 1 or more of the normalized cortical volume labels generated by FreeSurfer^25^. Furthermore, we excluded *n* = 3 scans from HC aged <15 years, in order to have a similar age range for FEP and HC groups (15–35 years). This left 74 FEP patients and 64 HC for the present study.

### Statistical analysis

#### Main analysis <whole sample>. The effect of age-by-diagnosis on CT/CSA/CV longitudinal changes at lobar level

To assess the effect of the age-by-diagnosis interaction on lobar PC of CT/CSA/CV, we used GLMs in which age at baseline, diagnosis, age-by-diagnosis, sex, TBV and site were included as independent variables, and lobar PC of CT/CSA/CV as dependent variables. We validated results using bootstrapping in 10.000 simulated datasets (with replacement) randomly selected from the original sample, and obtained mean effect sizes with 95% confidence intervals. Analyses were Bonferroni corrected, leading to a two-tailed alpha value of 0.004 [0.05/(4 lobes* 3 metrics (CT/CSA/CV))].

Before running GLM analyses, we explored the effect of two variables previously reported as potentially relevant for modulating CT/CSA/CV longitudinal changes in psychosis, namely antipsychotic intake over the follow-up (in patients) and premorbid IQ (in patients and HC). We also explored a potential non-linear effect of age at baseline as a quadratic term (in patients and HC). None of these covariates showed significant effects on lobar PC of CT/CSA/CV so we decided to exclude them from the final set of models (Supplementary Table 9).

#### Secondary analysis 1 <whole sample>. The effect of age-by-diagnosis on CT/CSA/CV longitudinal changes in temporal lobe regions

Subsequently, only in those lobes in which we found a significant age-by-diagnosis interaction effect (i.e., the temporal lobe), we explored the age-by-diagnosis interaction effect on the PC of CT/CSA/CV at a regional level (i.e., in the superior temporal gyrus, middle temporal gyrus, inferior temporal gyrus, fusiform gyrus, entorhinal cortex, parahippocampal gyrus, banks of the superior temporal sulcus, transverse temporal gyrus, and temporal pole). Age at baseline, diagnosis, age-by-diagnosis, sex, TBV and site were included as independent variables in the GLMs.

#### Secondary analysis 2 <whole sample>. The effect of diagnosis on temporal lobe CT at T1 and T2: separately in the adolescent FEP-HC, and adult FEP-HC samples

As a confirmatory post-hoc analysis, and only for those lobar metrics for which a significant age-by-diagnosis effect was found (CT of temporal lobe) we explored the effect of diagnosis (FEP vs. HC) on this metric at T1 and at T2, separately in an adolescent FEP-HC, and adult FEP-HC sample.

(i) In order to choose the optimal age cut-off value, we first stratified FEP cases and HC into several age cut-off pairs by establishing different age cut-off values (e.g., ≤18 years and >18 years, ≤19 years and >19 years, etc.). Then, using GLMs (same covariates and validation procedures as described for Main analysis), we explored, for CT of temporal lobe the effect of diagnosis for each “adolescent” and “adult” FEP-HC pair separately. We computed *t* values, and Cohen’s *d* values (effect sizes) with 95% confidence intervals for each diagnostic pair, and selected the age cut-off at which case-control differences became greatest (i.e., the largest effect size was observed). Supplementary Fig. 3 shows the effect sizes for FEP-HC comparisons in PC of temporal lobe CT, at different age cut-offs, for “adolescent” (age below the cut-off) and “adult” (age above the cut-off) individuals. Across all the age cut-off decisions, the largest effect size (i.e., the maximum absolute value for Cohen’s *d*) for the FEP–HC comparison in PC of temporal lobe CT was found at an age cut-off of 19 years. We selected the age cut-off of 19 years based on this criterion, but also on the basis that the sample size in each “age FEP-HC pair” was around *n* = 30 (to avoid violations of parametric model assumptions). We then defined a “≤19 year” subgroup (hereafter the “adolescent group”, *n* = 26 FEP and 24 HC) and a “>19 year” subgroup (hereafter the “adult group”, *n* = 48 FEP and 40 HC).

(ii) We used GLMs to assess the effect of diagnosis (FEP vs. HC) on temporal lobe CT at T1 and at T2, separately in an adolescent FEP-HC, and adult FEP-HC samples. Age at FEP, diagnosis, age-by-diagnosis, sex, TBV and site were included as independent variables in the GLMs. Validation procedures were the same as for Main analysis.

(iii) We also used repeated measures linear mixed models to assess, separately in the adolescent FEP-HC, and adult FEP-HC samples, diagnostic differences in the absolute change of temporal lobe CT (T2–T1). In these models, we set the time-point as a fixed factor and the subject’s ID as a random factor (intercept). We included age at FEP, sex, TBV and site as covariates and time between scans (in months) as an additional covariate. Validation procedures were the same as for Main analysis. To compare the goodness of fit of each model against a null model where the time-point effect was not included we used the likelihood ratio test and reported log-likelihood (LogLik) values.

#### Secondary analysis 3 <FEP sample>. Exploring the FEP group (adolescent or adult) whose temporal lobe CT measurements changed most over the follow-up period

We assessed which was the FEP group (adolescent or adult) whose temporal lobe CT measurement “changed most over the follow-up period”. First, separately for the “adolescent FEP-HC group” and for “the adult FEP-HC group”, we computed the “distance” of each patient’s temporal lobe CT at T1 to the mean of their age-matched HC group. Then, only for patients, we assessed the relationship between their “temporal lobe CT at T1 patient-control distance” and their PC of temporal lobe CT.

#### Secondary analysis 4 <FEP sample>. Clustering of FEP patients based on temporal lobar and regional CT

In a final step, we assessed whether CT of temporal lobe and CT of temporal lobe subregions could be used in a multivariate fashion to stratify the FEP sample (regardless of age) into meaningful biologically-driven subgroups. To determine the optimal number of clusters that were present in the imaging data, we used a *K* means clustering technique with the elbow method. We confirmed results with partitioning around medoids (PAM) using the optimum average silhouette width. For the identified FEP clusters, we conducted:

(i) Group comparisons in demographic and clinical variables at T1 and T2, as well as in those variables capturing “change”.

(ii) GLMs to assess the effect of cluster membership (cluster 1 vs. 2) on temporal lobe CT (either at T1 or PC). We included age at baseline scan, sex, TBV and site as covariates of no interest.

(iii) Repeated measures linear mixed models to assess cluster differences in temporal lobe CT absolute change (T2–T1). We set the time-point as a fixed factor and the subject’s ID as a random factor (intercept). We included age at baseline, sex, TBV and site as covariates and time between scans (in months) as an additional covariate. To compare the goodness of fit of each model against a null model where the time-point effect was not included we used the likelihood ratio test and reported log-likelihood (LogLik) values.

(iv) Separately for cluster 1 and cluster 2, GLMs to explore potential associations between PC of temporal CT and clinical variables of interest.

### Reporting summary

Further information on research design is available in the Nature Research Reporting Summary linked to this article.

## Supplementary information

Supplemental Material

REPORTING SUMMARY

## Data Availability

The data that support the findings of this study are available on request from the corresponding author (L.P-C.), subject to permission from the senior author of each site (M.J.C., A.G.P., E.V., J.C.F., A.L., M.B., M.P.), upon reasonable request.
