# Supplementary material for: Cortical thinning over two years after first-episode psychosis depends on age of onset
Source: Schizophrenia (Heidelb). 2022 Mar 11;8(1):20. doi: 10.1038/s41537-021-00196-7 (PMC8917180; doi:10.1038/s41537-021-00196-7)
Supplement: Supplementary file 1 — Supplemental Material [file 41537_2021_196_MOESM1_ESM.pdf]

**Supplementary Figure 1.**

**Association between PC of CT in four temporal regions and PANSS negative follow-up change score, separately for cluster 1 and cluster 2 FEP patients**

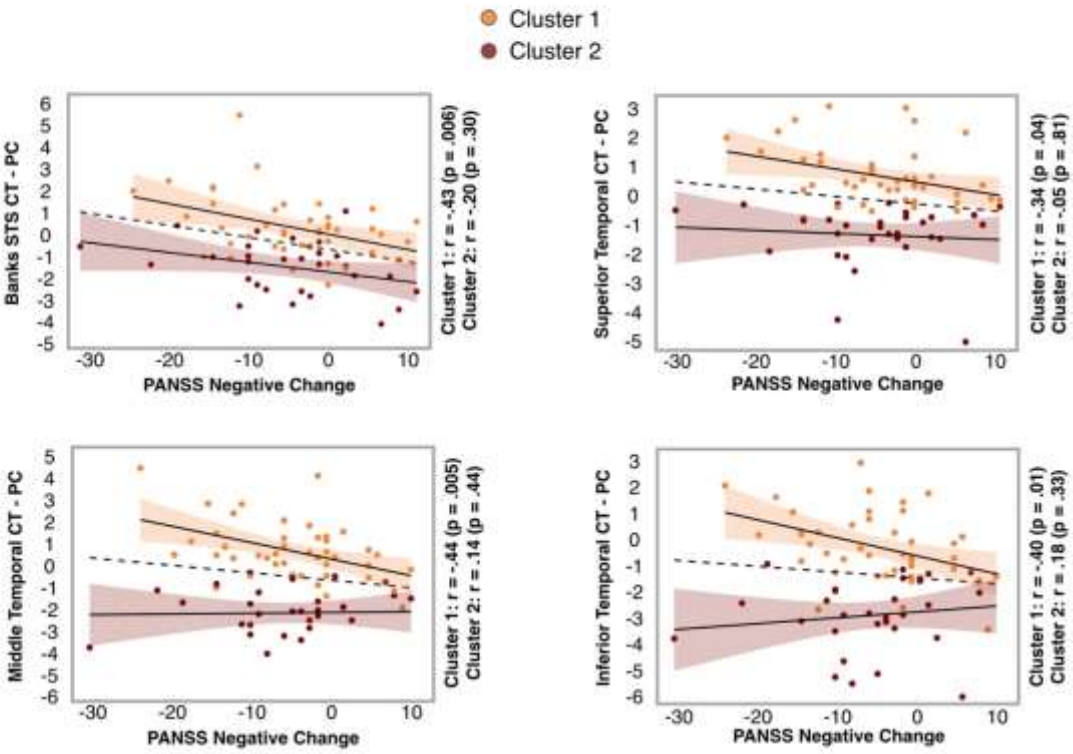


PC values < 0 indicate cortical thinning over follow-up; PC values > 0 indicate cortical thickening over follow-up. PANSS negative change score (T2-T1 score) < 0 indicate symptom improvement; PANSS negative change score > 0 (T2-T1 score) indicate symptom worsening (T2-T1 score).

Abbreviations: CT: cortical thickness; FEP: first-episode psychosis; PANSS: positive and negative syndrome scale; PC: percentage of change; STS: superior temporal sulcus

**Supplementary Figure 2.**

**Association between PC of CT in various temporal lobe ROIs and FEP patient's clinical variables, separately for the whole FEP sample (top) and for clusters 1 and 2 (bottom)**

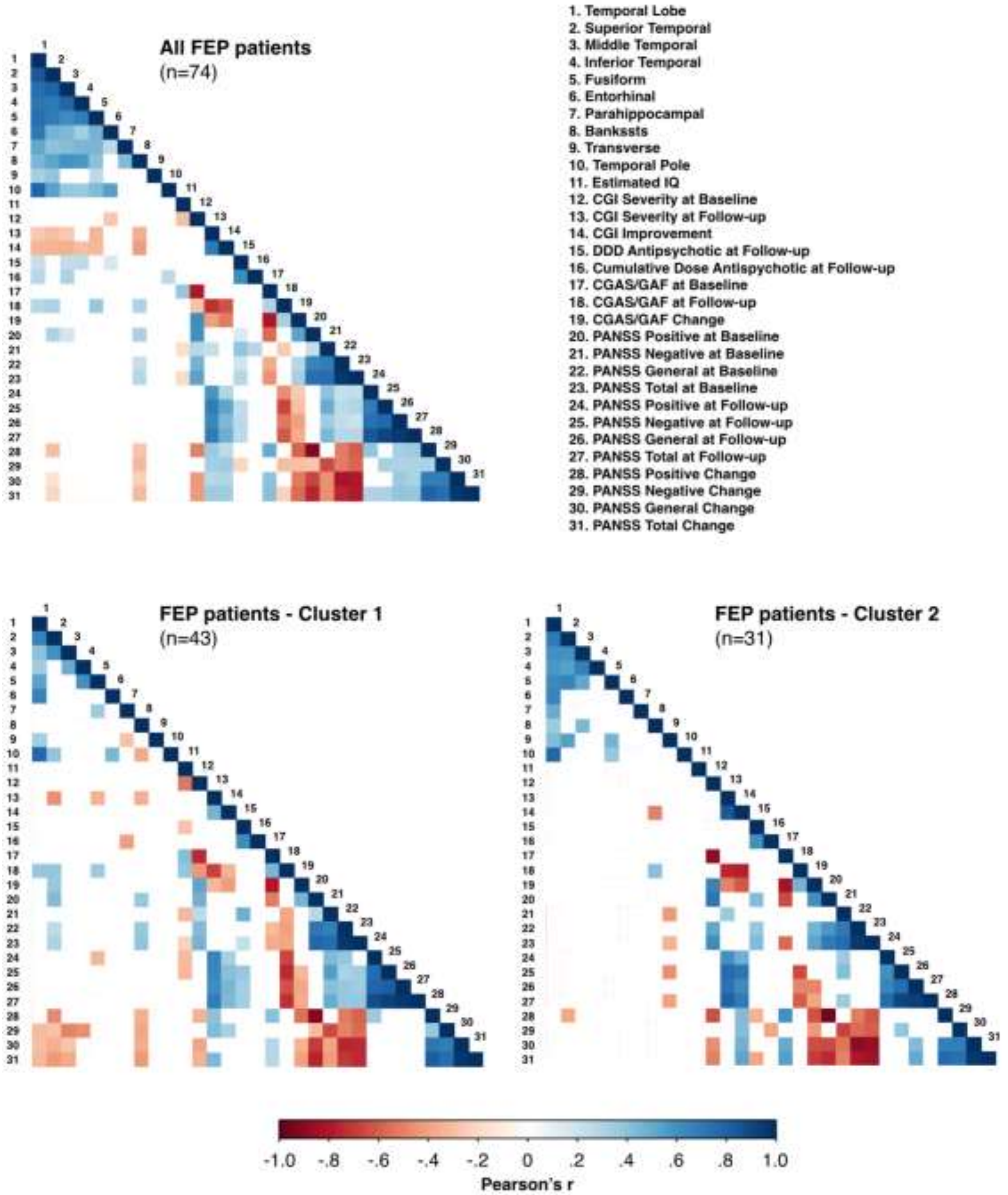


In the correlation matrix, only significant relationships ($p < .05$) are color-coded according to the color bar shown on the bottom.

Abbreviations: DDD: mean daily dose of antipsychotics, in chlorpromazine equivalents; CGAS: Children's Global Assessment Scale; CGI: Clinical Global impression scale ; CT: cortical thickness; FEP: first episode of psychosis; GAF: Global Assessment of Functioning scale; IQ: intelligence quotient; PANSS: positive and negative syndrome scale; PC: percentage of change; ROI: region of interest

**Supplementary Figure 3. Age cut-off selection procedure**

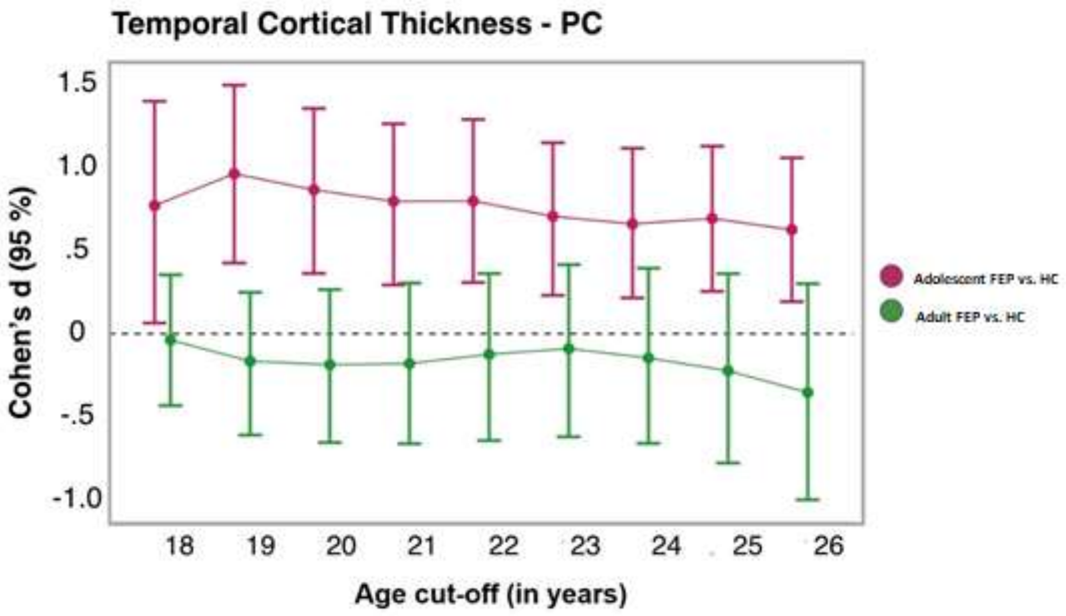


Effect sizes for FEP vs. HC comparisons in PC of temporal lobe CT, at different age cut-offs are shown, separately for “adolescent” and “adult” individuals. Adolescent” individuals= aged below cut-off. “Adult” individuals: aged above cut-off. The largest effect sizes were found at 19-year age cut-off.

Abbreviations: CT: cortical thickness; FEP: first episode psychosis; HC: healthy controls; PC: percentage of change

**Supplementary Table 1. Descriptives for lobar CT/CSA/CV at baseline (T1) and follow-up (T2) scans, and percentage of change (PC), and the independent effect of diagnosis and age at baseline scan**

| | | Baseline (T1) | | | | Follow-Up (T2) | | | | Percentage of change (PC) | | | |
|---|---|---|---|---|---|---|---|---|---|---|---|---|---|
| | | Mean (SD) | | Diagnosis effect * | Age at baseline effect * | Mean (SD) | | Diagnosis effect * | Age at baseline effect * | Mean (SD) | | Diagnosis effect * | Age at baseline effect * |
| | | FEP | HC | d-value (p-value) | r-value (p-value) | FEP | HC | d-value (p-value) | r-value (p-value) | FEP | HC | d-value (p-value) | r-value (p-value) |
| Cortical Thickness (CT) | Frontal | 2.67 (.10) | 2.64 (.11) | .294 (.093) | -.388 (.000004) | 2.67 (.10) | 2.65 (.10) | .152 (.384) | -.342 (.00005) | -.06 (1.10) | .21 (1.80) | -.188 (.283) | .090 (.302) |
| | Temporal | 2.93 (.10) | 2.92 (.13) | .020 (.909) | -.131 (.133) | 2.90 (.11) | 2.88 (.12) | .236 (.178) | -.053 (.540) | -.34 (1.35) | -.74 (1.76) | .270 (.123) | .079 (.367) |
| | Parietal | 2.53 (.08) | 2.52 (.10) | .171 (.328) | -.391 (.000003) | 2.49 (.08) | 2.49 (.10) | .0003 (.988) | -.367 (.00001) | -.80 (.93) | -.50 (1.29) | -.239 (.173) | .042 (.630) |
| | Occipital | 1.99 (.10) | 1.99 (.10) | -.082 (.639) | -.153 (.077) | 1.97 (.10) | 1.97 (.10) | -.097 (.578) | -.176 (.042) | -.47 (1.60) | -.50 (1.64) | -.004 (.984) | .020 (.819) |
| Cortical Surface Area (CSA) | Frontal | 64436 (6572) | 62380 (6376) | .183 (.295) | -.368 (.00001) | 64893 (6908) | 62448 (6663) | .256 (.143) | -.257 (.003) | .34 (1.13) | .08 (1.31) | .164 (.348) | .227 (.008) |
| | Temporal | 33182 (3238) | 32218 (3316) | .125 (.473) | -.245 (.005) | 34121 (3402) | 32838 (3320) | .277 (.114) | -.149 (.087) | 1.42 (1.35) | 1.01 (1.59) | .284 (.106) | .160 (.065) |
| | Parietal | 57119 (5652) | 55777 (5553) | .029 (.867) | -.341 (.00006) | 57676 (5781) | 55999 (5665) | .761 (.448) | -.284 (.0009) | .49 (1.07) | .22 (1.34) | .203 (.245) | .122 (.159) |
| | Occipital | 21739 (2240) | 20971 (2260) | .223 (.202) | -.143 (.100) | 21939 (2500) | 21040 (2382) | .258 (.140) | -.094 (.278) | .47 (1.59) | .19 (1.67) | .131 (.452) | .115 (.188) |
| Cortical Volume (CV) | Frontal | 190958 (19784) | 183629 (21327) | .324 (.065) | -.595 (<.000001) | 193252 (19872) | 186093 (22133) | .272 (.120) | -.537 (<.000001) | .66 (1.64) | .72 (2.20) | -.060 (.729) | .140 (.106) |
| | Temporal | 112536 (10530) | 108787 (12383) | .227 (.194) | -.420 (<.000001) | 113757 (10792) | 108879 (11921) | .328 (.032) | -.290 (.0007) | .56 (1.42) | .13 (2.31) | .212 (.226) | .213 (.013) |
| | Parietal | 157927 (15913) | 153436 (17460) | .119 (.497) | -.519 (<.000001) | 157580 (16215) | 153724 (17228) | .041 (.813) | -.489 (<.000001) | -.08 (1.54) | .14 (1.75) | -.143 (.410) | .099 (.257) |
| | Occipital | 48656 (5634) | 46894 (5489) | .177 (.311) | 0.224 (.009) | 48574 (6326) | 46606 (5369) | .207 (.237) | -.184 (.034) | -.08 (2.04) | -.23 (2.21) | .042 (.809) | .101 (.246) |

*Independent variables in each GLM: diagnosis (FEP vs. HC), age at baseline, sex, site and total brain volume (TBV). GLM results validated with bootstrapping in 10.000 simulated datasets (with replacement) randomly selected from the original sample, and Bonferroni-corrected leading to a two-tailed significant alpha value of 0.001 [0.05 / (3 metrics (CT, CSA, CV)) *3 time points (T1, T2, PC) *4 lobes)]; Abbreviations: FEP=first episode psychosis; HC=healthy controls

**Supplementary Table 2. Adolescent vs adult group comparison in demographic and clinical data, separately for FEP and HC samples**

| | FEP (N = 74) | Adolescent FEP (≤19y) n = 26 | Adult FEP (>19y) n = 48 | Cohen's d p-value | HC (N = 64) | Adolescent HC (≤19y) n = 24 | Adult HC (>19y) n = 40 | Cohen's d P value |
|---|---|---|---|---|---|---|---|---|
| Site, No (%) [1] | | | | | | | | |
| Barcelona | 40 (54.1) | 12 (46.2) | 28 (58.3) | d=-0.235 p=.316 | 35 (54.7) | 11 (45.8) | 24 (60.0) | d=-0.278 p=.270 |
| Madrid | 11 (14.9) | 9 (34.6) | 2 (4.2) | d=-0.895 p=.001 | 12 (18.8) | 7 (29.2) | 5 (12.5) | d=-0.423 p=.113 |
| Zaragoza | 15 (20.3) | 5 (19.2) | 10 (20.8) | d=-0.038 p=.870 | 11 (17.2) | 6 (25.0) | 5 (12.5) | d=-0.325 p=.305 |
| Vitoria | 8 (10.8) | 0 (0) | 8 (16.7) | d=-0.530 p=.044 | 6 (9.4) | 0 (0) | 6 (15.0) | d=-0.515 p=.076 |
| Age at T1 scan. Years, mean (SD) [range] | 23.2 (6.00) [15 - 35] | 17.0 (1.09) | 26.5 (4.79) | d=3.190 p<.001 | 23.7 (5.92) [15 - 35] | 17.5 (1.50) | 27.5 (4.11) | d=3.589 p<.001 |
| Sex. Male, No. (%) | 50 (67.6) | 17 (65.4) | 33 (68.8) | d=-0.069 p=.799 | 43 (67.2) | 20 (83.3) | 23 (57.5) | d=0.553 p=.033 |
| Time between T1 and T2 scans, months, mean (SD) [range] | 24.5 (2.5) [16 - 31] | 25.2 (2.3) [22 - 31] | 24.1 (2.6) [16 - 31] | d=-0.438 p=.070 | 23.9 (2.8) [20 - 32] | 23.8 (2.7) [20-32] | 24.0 (2.8) [20 - 31] | d=-0.774 p=.775 |
| Ethnicity. No, (%). | | | | | | | | |
| Caucasian | 68 (91.9) | 24 (92.3) | 44 (91.7) | d=-0.022 p=.999 | 58 (90.6) | 21 (87.5) | 37 (92.5) | d=-0.167 p=.664 |
| Hispanic | 3 (4.1) | 1 (3.8) | 2 (4.2) | d=-0.015 p=.999 | 3 (4.7) | 1 (4.2) | 2 (5.0) | d=-0.038 p=.999 |
| Other | 3 (4.1) | 1 (3.8) | 2 (4.2) | d=-0.015 p=.999 | 3 (4.7) | 2 (8.3) | 1 (2.5) | d=-0.270 p=.551 |
| Handedness, No. (%) | | | | | | | | |
| Right-handed | 59 (79.7) | 18 (69.2) | 41 (85.4) | d=-0.392 p=.098 | 53 (82.8) | 19 (79.2) | 34 (85.0) | d=-0.150 p=.734 |
| Left-handed | 8 (10.8) | 4 (15.4) | 4 (8.3) | d=-0.218 p=.440 | 7 (10.9) | 2 (8.3) | 5 (12.5) | d=-0.130 p=.702 |
| Mixed | 4 (5.4) | 3 (11.5) | 1 (2.1) | d=-0.408 p=.121 | 2 (3.1) | 1 (4.2) | 1 (2.5) | d=-0.093 p=.999 |
| Parental socioeconomic status [2] No. (%) | | | | | | | | |
| High – intermediate (vs low) | 53 (71.6) | 6 (23.1) | 8 (16.7) | d=-0.174 p=.457 | 54 (84.4) | 21 (87.5) | 33 (82.5) | d=-0.134 p=.731 |
| Premorbid IQ.[3] Score, mean (SD) [range] | 93.8 (16.9) [60 - 140] | 87.3 (20.5) [60 - 140] | 97.5 (13.5) [70 - 120] | d=-0.553 p=.029 | 108.3 (11.9) [85 - 145] | 105.2 (10.5 [85 - 125] | 110.1(12.4) [90-145] | d=-0.154 p=.115 |
| Diagnostic subgroup [4], No (%) | | | | | -- | -- | -- | -- |
| SSD | 38 (51.4) | 15 (57.7) | 23 (47.9) | d=-0.188 p=.576 | | | | |
| AFP | 17 (23.0) | 8 (30.8) | 9 (19.8) | d=-0.275 p=.241 | | | | |
| Other psychoses | 19 (25.7) | 3 (11.5) | 16 (33.3) | d=-0.491 p=.040 | | | | |
| Illness severity (CGI-S) at T1, mean (SD) | 4.40 (1.3) | 4.88 (1.4) | 4.13 (1.1) | d=-0.608 p=.015 | -- | -- | -- | -- |
| CGAS/GAF score at T1, mean (SD) | 53.5 (23.1) | 43.7 (24.7) | 58.9 (20.5) | d= 0.689 p=.006 | 93.3 (5.7) | 92.3 (6.2) | 93.9 (5.5) | d= 0.277 p=.296 |
| Cumulative antipsychotic dose at T2 [5], mg, median [range] | 301.042 [0 – 1.088.332] | 289.591 [0 – 881.288] | 308.198 [0 – 1.088.332] | d=0.084 p=.742 | -- | -- | -- | -- |

In all cells, % refers to percentages (within columns) of participants for whom information was available. For qualitative variables, Chi-square ($\chi 2$) or Fisher tests were used. For quantitative variables, t-tests or Mann-Whitney U tests were used.

[1] Note: two sites of the PEPs-Imaging cross-sectional study did not participate in the longitudinal branch of the study

[2] Socioeconomic status (SES) defined with the Hollingshead-Redlich scale. Individuals were categorized into a low (SES 4 to 5) vs intermediate-high (SES 1 to 3) parental SES subgroup

[3] Estimated with the vocabulary subtest of the WISC-IV or WAIS-III for subjects under and over 16 years of age

[4] SSD: 24-month follow-up diagnosis of schizophrenia spectrum disorder (schizophrenia, schizophreniform, or schizoaffective disorder); AFP: 24-month follow-up diagnosis of affective psychosis (type I bipolar disorder or major depressive disorder with psychotic symptoms); Other psychoses: 24-month follow-up diagnosis of brief reactive psychosis, delusional disorder, substance-induced psychotic disorder, and psychotic disorder not otherwise specified

[5] In chlorpromazine equivalents: 100 mg chlorpromazine = about 1.5 mg risperidone / 5 mg olanzapine / 150 mg quetiapine

Abbreviations: CGAS: Children's Global Assessment Scale; CGI-S: Clinical Global impression-Severity scale; FEP: first episode of psychosis; GAF: Global Assessment of Functioning scale; HC: healthy control; IQ: intelligence quotient; T1: baseline scan visit; T2: follow-up scan visit

**Supplementary Table 3. Descriptives for lobar and regional temporal CT at T1 and PC for cluster 1 and cluster 2 FEP patients, and the effect of cluster membership on CT at T1 and PC**

| | | Cluster 1, n = 43 (mean) | Cluster 2, n = 31 (mean) | Cluster Membership d-value | Cluster Membership p-value |
|---|---|---|---|---|---|
| **Cortical Thickness at T1** | **Temporal Lobe** | 2.889 | 2.973 | .629 | .015 |
| | **Superior Temporal** | 2.850 | 2.917 | .429 | .077 |
| | **Middle Temporal** | 2.912 | 3.023 | .659 | .008 |
| | **Inferior Temporal** | 2.792 | 2.909 | .857 | .0006 |
| | **Fusiform** | 2.715 | 2.777 | .536 | .028 |
| | **Entorhinal** | 3.338 | 3.413 | .318 | .188 |
| | **Parahippocampal** | 2.681 | 2.827 | .549 | .025 |
| | **Banks STS** | 2.572 | 2.643 | .328 | .175 |
| | **Transverse Temporal** | 2.536 | 2.613 | .320 | .185 |
| | **Temporal Pole** | 3.594 | 3.626 | .097 | .688 |
| **PC of Cortical Thickness** | **Temporal Lobe** | .028 | -.187 | -2.01 | <.0000005 |
| | **Superior Temporal** | .078 | -.103 | -1.987 | <.0000005 |
| | **Middle Temporal** | .063 | -.183 | -2.249 | <.0000005 |
| | **Inferior Temporal** | .025 | -.226 | -1.903 | <.0000005 |
| | **Fusiform** | .038 | -.163 | -1.510 | <.0000005 |
| | **Entorhinal** | .098 | -.211 | -.919 | .0003 |
| | **Parahippocampal** | .014 | -.195 | -1.245 | .000002 |
| | **Banks STS** | .018 | -.145 | -1.296 | .0000009 |
| | **Transverse Temporal** | -.052 | -.074 | -.137 | .57 |
| | **Temporal Pole** | .017 | -.299 | -.770 | .002 |

PC values < 0 indicate cortical thinning. PC values > 0 indicate cortical thickening. T1: baseline scan

Abbreviations: FEP: first episode psychosis; PC: percentage of change; STS: superior temporal sulcus

**Supplementary Table 4. Repeated measures linear mixed models. Differences between cluster 1 and cluster 2 FEP patients in the absolute longitudinal (T2-T1) change of lobar and regional temporal CT**

| | | T1 (mean) | T2 (mean) | Time point LogLik | Time point p– value |
|---|---|---|---|---|---|
| **Cluster 1** n = 43 **Cortical Thickness (mm)** | **Temporal Lobe** | 2.889 | 2.907 | 2.969 | .084 |
| | **Superior Temporal** | 2.850 | 2.903 | 23.655 | p<.0001 |
| | **Middle Temporal** | 2.912 | 2.954 | 11.486 | .0007 |
| | **Inferior Temporal** | 2.792 | 2.805 | .993 | .319 |
| | **Fusiform** | 2.715 | 2.738 | 4.397 | .036 |
| | **Entorhinal** | 3.338 | 3.411 | 4.899 | .027 |
| | **Parahippocampal** | 2.681 | 2.689 | .253 | .615 |
| | **Banks STS** | 2.572 | 2.583 | .802 | .371 |
| | **Transverse Temporal** | 2.536 | 2.497 | 3.583 | .058 |
| | **Temporal Pole** | 3.594 | 3.584 | .031 | .861 |
| **Cluster 2** n = 31 **Cortical Thickness (mm)** | **Temporal Lobe** | 2.973 | 2.841 | 44.238 | p<.0001 |
| | **Superior Temporal** | 2.917 | 2.845 | 25.275 | p<.0001 |
| | **Middle Temporal** | 3.023 | 2.890 | 45.182 | p<.0001 |
| | **Inferior Temporal** | 2.909 | 2.751 | 45.020 | p<.0001 |
| | **Fusiform** | 2.777 | 2.669 | 27.540 | p<.0001 |
| | **Entorhinal** | 3.413 | 3.237 | 12.507 | .0004 |
| | **Parahippocampal** | 2.827 | 2.694 | 35.210 | p<.0001 |
| | **Banks STS** | 2.643 | 2.549 | 32.481 | p<.0001 |
| | **Transverse Temporal** | 2.613 | 2.564 | 6.544 | .011 |
| | **Temporal Pole** | 3.626 | 3.358 | 16.449 | p<.0001 |

Repeated measures linear mixed models. Time point set as fixed factor and subject's ID as random factor (intercept). Age at baseline scan, sex, TBV, time between scans (in months) and site as covariates of no interest. T1: baseline scan; T2: follow-up scan

Abbreviations: CT: cortical thickness; FEP: first episode psychosis; STS: superior temporal sulcus

**Supplementary Table 5. FEP clusters. Demographic and clinical characteristics**

| | FEP Cluster 1 (n = 43) | FEP Cluster 2 (n = 31) | Cohen's d; p-value |
|---|---|---|---|
| Site, No (%) [1] | | | |
| Barcelona | 24 (55.8) | 16 (51.6) | d=0.337; p=.563 |
| Madrid | 8 (18.6) | 3 (9.7) | |
| Zaragoza | 7 (16.3) | 8 (25.8) | |
| Vitoria | 4 (9.3) | 4 (12.9) | |
| Age at T1 scan. Years, mean (SD) [range] | 24.5 (6.6) [15 - 35] | 21.3 (4.6) [15 - 34] | d=0.591; p=.014 |
| Sex. Male, No. (%) | 32 (74.4) | 18 (58.1) | d=0.350; p=.138 |
| Diagnostic subgroup [2], No (%) | | | |
| SSD | 23 (53.5) | 15 (48.4) | d=0.251; p=.565 |
| AFP | 8 (18.6) | 9 (29.0) | |
| Other psychoses | 12 (27.9) | 7 (22.6) | |
| Duration of FEP until T1 scan, months, median [range] | 5 [0- 13] | 3 [1 – 13] | d=0.036; p=.880 |
| Time between T1 and T2 scans, months, median [range] | 24 [16 - 31] | 24 [21 - 28] | d=0.404; p=.092 |
| Ethnicity. No. (%) | | | |
| Caucasian | 41 (95.3) | 27 (87.1) | d=0.302; p=.439 |
| Hispanic | 1 (2.3) | 2 (6.5) | |
| Other | 1 (2.3) | 2 (6.5) | |
| Parental socioeconomic status [3] No. (%) | | | |
| High – intermediate (vs. low) | 31 (72.1) | 22 (71.1) | d=0.024; p=.916 |
| Handedness, No. (%) | | | |
| Right-handed | 32 (74.4) | 27 (87.1) | d=0.209; p=.672 |
| Left-handed | 5 (11.6) | 3 (9.7) | |
| Mixed | 3 (7.0) | 1 (3.2) | |
| Premorbid IQ [4] Score, mean (SD) [range] | 96.5 (15.3) [60 - 120] | 90.3 (18.6) [65 - 140] | d=0.362; p=.129 |
| Illness severity, mean (SD) | | | |
| CGI-S at T1 | 4.47 (1.1) | 4.30 (1.5) | d=0.126; p=.594 |
| CGI-S at T2 | 2.5 (1.2) | 2.9 (1.5) | d=0.300; p=.207 |
| CGAS/GAF score, mean (SD) | | | |
| CGAS/GAF at T1 | 55.1 (20.6) | 51.5 (26.4) | d=0.146; p=.538 |
| CGAS/GAF at T2 | 77.8 (13.1) | 71.6 (16.5) | d=0.414; p=.083 |
| Antipsychotic treatment, N (%) | | | |
| At T1 | 41 (95.3), 100% SGA | 27 (87.1), 100% SGA | d=0.146; p=.538 |
| At T2 | 22 (62.9), 100% SGA | 22 (73.3), 100% SGA | |
| Cumulative antipsychotic dose at T2 [5], mg, median [range] | 360 813 [0 – 1 088 332] | 231 308 [0 – 556 887] | d=0.577; p=.016 |
| Lithium treatment, N (%) | | | |
| At T1 | 2 (4.7) | 6 (19.4) | d=0.389; p=.167 |
| At T2 | 3 (8.6) | 7 (23.3) | |

In all cells, % refers to percentages (within columns) of participants for whom information was available. For qualitative variables, Chi-square (χ2) or Fisher tests were used. For quantitative variables, t-tests or Mann-Whitney U tests were used.

[1] Note: two sites of the PEPs-Imaging cross-sectional study did not participate in the longitudinal branch of the study

[2] SSD: 24-month follow-up diagnosis of schizophrenia spectrum disorder (schizophrenia, schizophreniform, or schizoaffective disorder); AFP: 24-month follow-up diagnosis of affective psychosis (type I bipolar disorder or major

depressive disorder with psychotic symptoms); Other psychoses: 24-month follow-up diagnosis of brief reactive psychosis, delusional disorder, substance-induced psychotic disorder, and psychotic disorder not otherwise specified
[2] Socioeconomic status (SES) defined with the Hollingshead-Redlich scale. Individuals were categorized into a low (SES 4 to 5) vs intermediate-high (SES 1 to 3) parental SES subgroup

[4] Estimated with the vocabulary subtest of the WISC-IV or WAIS-III for subjects under and over 16 years of age

[5] In chlorpromazine equivalents: 100 mg chlorpromazine = about 1.5 mg risperidone / 5 mg olanzapine / 150 mg quetiapine

Abbreviations: CGAS: Children's Global Assessment Scale; CGI-S: Clinical Global impression-Severity scale; FEP: first episode of psychosis; GAF: Global Assessment of Functioning scale; IQ: intelligence quotient; SGA: second-generation antipsychotics; T1: baseline scan visit; T2: follow-up scan visit

**Supplementary Table 6. Scanner platforms of the PEPs-Longitudinal-Imaging study and acquisition parameters by site**

| Site number | Site | Scanner | | Orientation | Voxel size ($mm^3$) | FOV ($mm^2$) | Slices | Acquisition Matrix | TR (ms.) | TE (ms.) | Inversion Time (ms.) | Flip Angle | Sequence |
|---|---|---|---|---|---|---|---|---|---|---|---|---|---|
| 1 | Barcelona | SIEMENS-TRIO TIM (3T) | T1 | Sagittal | 1x1x1 | 256x256 | 240 | 256x256 | 2300 | 2.98 | 900 | 9 | Gradient Echo/IR 3D |
| 2 | Madrid | PHILIPS-INTERA (I.5T) | T1 | Sagittal | 1x0.94x0.94 | 240x240 | 175 | 240x240 | 25 | 9.18 | - | 30 | Gradient Echo (FFE 3D) |
| 3 | Vitoria | SIEMENS-AVANTO (I.5T) | T1 | Sagittal | 1x0.95x0.95 | 243x243 | 176 | 256x256 | 25 | 9.21 | - | 30 | Gradient Echo 3D |
| 4 | Zaragoza | GENERAL ELECTRIC-SIGNA EXCITE (I.5T) | T1 | Sagittal | 1x0.94x0.94 | 240x240 | 156 | 256x256 | 9,28 | 1x90 | 450 | 20 | Gradient Echo/IR 3D |

**Supplementary Table 7. Regional ROIs under study (in alphabetical order)**

| | |
|---|---|
| Caudal anterior cingulate | Pars opercularis |
| Caudal middle frontal | Pars orbitalis |
| Cuneus | Pars triangularis |
| Entorhinal | Pericalcarine |
| Fusiform | Postcentral |
| Banks | Posterior cingulate |
| Inferior parietal | Precentral |
| Inferior temporal | Precuneus |
| Insula | Rostral anterior cingulate |
| Isthmus cingulate | Rostral middle frontal |
| Lateral occipital | Superior frontal |
| Lateral orbitofrontal | Superior parietal |
| Lingual | Superior temporal |
| Medial orbitofrontal | Supramarginal |
| Middle temporal | Frontal pole |
| Parahippocampal | Temporal pole |
| Paracentral | Transverse temporal |

**Supplementary Table 8a. Follow-up "completers" vs "non-completers" in the PEP-Imaging study. Demographic and clinical variables at baseline**

| | FEP Patients | | | Healthy Controls | | |
|---|---|---|---|---|---|---|
| | Non-completers (N=99) | Completers (N=76) | *Cohen's d; p*-value | Non-completers (N=81) | Completers (N=67) | *Cohen's d; p*-value |
| Site, No (%) | | | | | | |
| Barcelona | 63 (63.6) | 42 (55.3) | d=0.313; p=.243 | 44 (54.3) | 34 (54.0) | d=0.313; p=.617 |
| Madrid | 14 (14.1) | 13 (17.1) | | 21 (25.9) | 12 (19.0) | |
| Zaragoza | 9 (9.1) | 14 (18.4) | | 9 (11.1) | 11 (17.5) | |
| Vitoria | 13 (13.1) | 7 (9.2) | | 7 (8.6) | 6 (9.5) | |
| Age at T1 scan. Years, mean (SD) [range] | 22.9 (6.3) [12 – 35] | 23.7 (5.8) [15 - 35] | d=0.130; p=.395 | 23.6 (6.3) [10 - 35] | 24.4 (5.9) [15 - 35] | d=0.125; p=.452 |
| Sex. Male, No. (%) | 66 (66.7) | 51 (67.1) | d=0.009; p=.951 | 46 (56.8) | 43 (68.3) | d=0.233; p=.160 |
| Diagnostic subgroup, No (%) | | | | | | |
| SSD | 50 (50.5) | 37 (48.7) | d=0.366; p=.059 | -- | -- | -- |
| AFP | 12 (12.1) | 19 (25.0) | | | | |
| Other psychoses | 37 (37.4) | 20 (26.3) | | | | |
| Duration of FEP until T1 scan, months, median [range] | 4.5 [0-16] | 3 [0-13] | d=0.179; p=.388 | --- | --- | --- |
| Ethnicity. No. (%) | | | | | | |
| Caucasian | 81 (81.8) | 69 (90.8) | d=0.276; p=.196 | 70 (86.4) | 58 (92.1) | d=0.179; p=.560 |
| Hispanic | 5 (5.1) | 3 (3.9) | | 7 (8.6) | 3 (4.8) | |
| Other | 13 (13.1) | 4 (5.3) | | 4 (4.9) | 2 (3.2) | |
| Parental socioeconomic status[b] No. (%) | | | | | | |
| Low | 54 (54.5) | 24 (31.6) | d=0.471; p=.002 | 27 (33.3) | 9 (14.3) | d=0.441; p=.009 |
| High-intermediate | 45 (45.5) | 52 (68.4) | | 54 (66.7) | 54 (85.7) | |
| Handedness, No. (%) | | | | | | |
| Right-handed | 76 (88.4) | 61 (83.6) | d=0.239; p=.293 | 70 (90.9) | 52 (85.2) | d=0.183; p=.541 |
| Left-handed | 9 (10.5) | 8 (11.0) | | 6 (7.8) | 7 (11.5) | |
| Mixed | 1 (1.2) | 4 (5.5) | | 1 (1.3) | 2 (3.3) | |
| Premorbid IQ[c] Score, mean (SD) [range] | 92.9 (14.8) [60 - 130] | 93.4 (17.0) [60 - 140] | d=0.028; p=.854 | 105.7 (14.9) [75 - 145] | 108.5 (11.8) [85-145] | d=0.202; p=.222 |
| Illness severity (CGI-S), mean (SD) [range] | 4.5 (1.07) [1 - 7] | 4.4 (1.3) [1 - 7] | d=0.121; p=.430 | --- | --- | --- |
| CGAS/GAF score, mean (SD) [range] | 49.5 (19.6) [15 - 95] | 54.2 (23.2) [15 - 90] | d=0.231; p=.129 | 93.6 (5.4) [80-100] | 93.5 (5.8) [80-100] | d= 0.308; p=.758 |

In all cells, % refers to percentages (within columns) of participants for whom information was available. For qualitative variables, Chi-square ($\chi 2$) or Fisher tests were used. For quantitative variables, t-tests or Mann-Whitney U tests were used. Abbreviations: CGAS: Children's Global Assessment Scale; CGI-S: Clinical Global impression-Severity scale; FEP: first episode of psychosis; GAF: Global Assessment of Functioning scale; IQ: intelligence quotient; T1: baseline scan visit

**Supplementary Table 8b. Two-year “completers” vs. “non-completers” in the PEP-Imaging study. Global brain measurements at baseline**

| | FEP Patients | | | Healthy Controls | | |
|---|---|---|---|---|---|---|
| | Non-completers (N=99) | Completers (N=76) | *Cohen’s d; p*-value | Non-completers (N=81) | Completers (N=67) | *Cohen’s d; p*-value |
| Total Brain Volume ($mm^3$)<br>*=Gray matter + white matter volume* | 1 010 734 (114 853) | 1 014 755 (111 777) | d=0.035; p=.817 | 1 026 303 (106 670) | 1 049 419 (107 034) | d=0.213; p=.200 |
| Total Gray Matter Volume ($mm^3$) | 531 604 (64 179) | 533 704 (61 440) | d=0.033; p=.827 | 538 153 (66 058) | 552 151 (51 975) | d=0.228; p=.169 |
| Whole brain Mean Cortical Thickness (mm) | 2.60 (0.11) | 2.62 (0.11) | d=0.183; p=.232 | 2.62 (0.11) | 2.64 (0.09) | d=0.105; p=.526 |
| Whole brain Surface Area ($mm^2$) | 169 630 (19 475) | 168 615 (18 639) | d=0.053; p=.728 | 170 668 (18 472) | 173 697 (17 191) | d=0.166; p=.316 |

Abbreviations: FEP: first episode of psychosis

**Supplementary Table 9. Effect of cumulative antipsychotic dose at follow-úp, estimated IQ and age at baseline squared on the PC of CT/CSA/CV at lobar level**

| | | Dependent Variable PC | Cumulative antipsychotic dose r – value | p – value |
|---|---|---|---|---|
| Independent Variables<br>Age at baseline<br>Sex<br>Site<br>Total Brain Volume<br>Cumulative antipsychotic dose at follow-up<br><br>**Analysis applies to FEP sample (n= 74)* | Cortical Thickness PC | Frontal | .134 | .123 |
| | | Parietal | .125 | .150 |
| | | Temporal | .143 | .102 |
| | | Occipital | .046 | .597 |
| | Cortical Surface Area PC | Frontal | -.004 | .967 |
| | | Parietal | -.030 | .732 |
| | | Temporal | -.014 | .869 |
| | | Occipital | -.097 | .267 |
| | Cortical Volume PC | Frontal | .088 | .313 |
| | | Parietal | .114 | .189 |
| | | Temporal | .071 | .418 |
| | | Occipital | -.037 | .675 |

| | | Dependent Variable PC | Estimated IQ r – value | p – value |
|---|---|---|---|---|
| Independent Variables<br>Age at baseline<br>Sex<br>Site<br>Total Brain Volume<br>Estimated IQ<br><br>**Analysis applies to whole sample (n= 74 FEP, 64 HC)* | Cortical Thickness PC | Frontal | -. 069 | .435 |
| | | Parietal | - .081 | .360 |
| | | Temporal | .140 | .111 |
| | | Occipital | -.065 | .463 |
| | Cortical Surface Area PC | Frontal | -.102 | .248 |
| | | Parietal | -.062 | .487 |
| | | Temporal | .107 | .224 |
| | | Occipital | .144 | .101 |
| | Cortical Volume PC | Frontal | -.132 | .134 |
| | | Parietal | -.130 | .140 |
| | | Temporal | .166 | .057 |
| | | Occipital | .039 | .655 |

| | | Dependent Variable: PC | Age at baseline squared r – value | p – value |
|---|---|---|---|---|
| Independent Variables<br>Age at baseline<br>Sex<br>Site<br>Total Brain Volume<br>Age at baseline squared<br><br>**Analysis applies to whole sample (n= 74 FEP, 64 HC)* | Cortical Thickness PC | Frontal | .163 | .063 |
| | | Parietal | .127 | .147 |
| | | Temporal | .103 | .242 |
| | | Occipital | .014 | .874 |
| | Cortical Surface Area PC | Frontal | -.038 | .660 |
| | | Parietal | -.165 | .060 |
| | | Temporal | -.200 | .022 |
| | | Occipital | .125 | .153 |
| | Cortical Volume PC | Frontal | .064 | .466 |
| | | Parietal | -.049 | .578 |
| | | Temporal | -.100 | .255 |
| | | Occipital | - .051 | .562 |

Abbreviations: CT : cortical thickness; CSA: cortical surface area; CV: cortical volume; FEP: first-episode psychosis; IQ: intelligence quotient; PC: percentage of change
